# Unbiased signal equation for quantitative magnetization transfer mapping in balanced steady‐state free precession MRI

**DOI:** 10.1002/mrm.28940

**Published:** 2021-07-31

**Authors:** Fritz M. Bayer, Michael Bock, Peter Jezzard, Alex K. Smith

**Affiliations:** ^1^ Wellcome Centre for Integrative Neuroimaging FMRIB Division Nuffield Department of Clinical Neurosciences University of Oxford Oxford UK; ^2^ D‐BSSE ETH Zurich Basel Switzerland; ^3^ Department of Radiology Medical Physics Medical Center—University of Freiburg Freiburg Germany; ^4^ Faculty of Medicine University of Freiburg Freiburg Germany

**Keywords:** balanced SSFP, magnetization transfer, quantitative imaging

## Abstract

**Purpose:**

Quantitative magnetization transfer (qMT) imaging can be used to quantify the proportion of protons in a voxel attached to macromolecules. Here, we show that the original qMT balanced steady‐state free precession (bSSFP) model is biased due to over‐simplistic assumptions made in its derivation.

**Theory and Methods:**

We present an improved model for qMT bSSFP, which incorporates finite radiofrequency (RF) pulse effects as well as simultaneous exchange and relaxation. Furthermore, a correction relating to finite RF pulse effects for sinc‐shaped excitations is derived. The new model is compared to the original one in numerical simulations of the Bloch‐McConnell equations and in previously acquired in vivo data.

**Results:**

Our numerical simulations show that the original signal equation is significantly biased in typical brain tissue structures (by 7%‐20%), whereas the new signal equation outperforms the original one with minimal bias (<1%). It is further shown that the bias of the original model strongly affects the acquired qMT parameters in human brain structures, with differences in the clinically relevant parameter of pool‐size‐ratio of up to 31%. Particularly high biases of the original signal equation are expected in an MS lesion within diseased brain tissue (due to a low T2/T1‐ratio), demanding a more accurate model for clinical applications.

**Conclusion:**

The improved model for qMT bSSFP is recommended for accurate qMT parameter mapping in healthy and diseased brain tissue structures.

## 1 INTRODUCTION

Quantitative magnetization transfer (qMT) imaging can be used to quantify the proportion of protons in a voxel attached to macromolecules. qMT has shown considerable promise for characterizing myelin‐related diseases, such as multiple sclerosis. Due to a high signal‐to‐noise ratio and short acquisition times, balanced steady‐state free precession (bSSFP) acquisition modules have become a popular method for quantifying MT parameters.^1^, ^2^, ^3^ However, the derivation of the qMT bSSFP signal equation is based on two major assumptions, which limit its generality and accuracy.

First, it is assumed that magnetization relaxation and the spin exchange between the free and macromolecular pool (MT) can be modeled as independent processes. This implies that the continuous phenomenon of MT has an instantaneous effect on the magnetization. Although the separation of exchange and relaxation simplifies the derivation of the original qMT bSSFP signal equation, this assumption does not accurately describe the physical nature of MT, as these effects happen simultaneously.

Furthermore, the originally proposed signal equation assumes an instantaneous rotation of magnetization by the RF pulse. Bieri and Scheffler have shown^4^, ^5^, ^6^, ^7^ that this assumption does not accurately describe the finite nature of an RF pulse in bSSFP due to an overestimation of transverse relaxation. While this effect is negligible for short pulse durations $\frac{T_{\mathrm{RF}}}{\mathrm{TR}}\ll 1$, a significant bias is introduced if that condition is not satisfied.^4^ In conventional bSSFP (non‐qMT), this bias can amount to 10% (α ∼ $90^{\circ }$, T2/T1 ≪ 1).^4^, ^5^ As qMT bSSFP is based on a stepwise variation of the RF pulse duration, this condition is certainly not met in the original qMT bSSFP acquisition scheme, where the ratio $\frac{T_{\mathrm{RF}}}{\mathrm{TR}}$ can be as high as 0.44.^1^, ^8^ A correction to this bias has been proposed for Gaussian pulse shapes, which are, however, not commonly used in qMT bSSFP, where a sinc pulse is more typically used.^1^, ^2^, ^3^

Here, we present an improved signal equation for qMT bSSFP, which incorporates finite pulse effects as well as simultaneous exchange and relaxation. A correction to finite RF pulse effects for sinc‐shaped excitations is derived. By means of numerical simulations of the Bloch‐McConnell equations, it is demonstrated that the original signal equation is significantly biased in typical brain tissue structures. Additionally, this bias is strongly dependent on the time‐bandwidth (TBW) product for sinc pulses; thus, a framework to minimize this bias is presented.

## 2 THEORY

In this section, a new qMT bSSFP signal equation is derived allowing for simultaneous magnetization exchange and relaxation, and correcting for the instantaneous rotation by the RF pulse. To model the magnetization dynamics, a single bSSFP acquisition cycle of duration *TR* is considered, that is repeated until steady state is reached. Each cycle can be split into two epochs:

- Excitation by the RF pulse,
- Free relaxation (including spin information exchange between pools).

To derive the magnetization at steady state, each epoch can be modeled independently and subsequently unified by the steady‐state condition.

Analogous to the original derivation,^1^ the excitation by the RF pulse (Epoch I) is initially assumed to be instantaneous $T_{\mathrm{RF}}\to 0$ (correction follows below). Thus, the magnetization state is instantly rotated at t=nTR, $n\in N_{0}$, which is described by the following formalism

$$\begin{array}{c} M\left(t^{\prime }=nTR\right)=\left\{\begin{array}{cc} M^{‐}\left(n\right) & \text{before rotation via the RF pulse}\\ M^{+}\left(n\right) & \text{after rotation via the RF pulse} \end{array}\right. \end{array}$$

This convention was established by Freeman^9^ and is commonly used in bSSFP. The RF pulse leads to a rotation of the free‐pool magnetization around the x‐axis and can therefore be modeled via the rotation matrix $R_{x}\left(\alpha \right)$, representing a clockwise rotation in the x‐plane with angle α for an anticlockwise polarized RF field. Simultaneously, the pulse saturates the macromolecular pool, which can be modeled using the mean saturation rate ⟨W(Δ→0)⟩. Thus, the operator, representing the action of the pulse on the magnetization $M={\left[M_{\mathrm{xf}},M_{\mathrm{yf}},M_{\mathrm{zf}},M_{\mathrm{zm}}\right]}^{T}$, is given by

(1) $$\begin{array}{c} R_{x}\left(\alpha ,t\right)=\left[\begin{array}{cccc} 1 & 0 & 0 & 0\\ 0 & \cos\alpha & \sin\alpha & 0\\ 0 & ‐\sin\alpha & \cos\alpha & 0\\ 0 & 0 & 0 & e^{‐\langle W(\Delta \to 0)\rangle t} \end{array}\right] \end{array}$$

satisfying the relation

(2) $$\begin{array}{c} M^{‐}\left(n\right)=R_{x}M^{+}\left(n\right) \end{array}$$

The mean saturation rate ⟨W(Δ)⟩ used in this derivation is equivalent to the one proposed in the work by Gloor and is described in detail elsewhere.^1^

During free relaxation (Epoch II), the magnetization $M={\left[M_{\mathrm{xf}},M_{\mathrm{yf}},M_{\mathrm{zf}},M_{\mathrm{zm}}\right]}^{T}$ can be modeled by solving the Bloch‐McConnell Equations in the unperturbed case

(3) $$\begin{array}{c} \frac{dM(t^{\prime })}{dt^{\prime }}=\left[\begin{array}{cccc} ‐R_{2f} & 0 & 0 & 0\\ 0 & ‐R_{2f} & \omega_{1} & 0\\ 0 & ‐\omega_{1} & ‐(R_{1f}+k_{\mathrm{fm}}) & k_{\mathrm{mf}}\\ 0 & 0 & k_{\mathrm{fm}} & ‐(R_{1m}+k_{\mathrm{fm}}) \end{array}\right]M\left(t^{\prime }\right)+\left[\begin{array}{c} 0\\ 0\\ R_{1f}M_{0f}\\ R_{1m}M_{0f}F \end{array}\right] \end{array}$$

where $t^{\prime }=t‐n\cdot TR$ with $n\in N_{0}$ is the time of the nth acquisition cycle and the magnetization is $M={\left[M_{\mathrm{xf}},M_{\mathrm{yf}},M_{\mathrm{zf}},M_{\mathrm{zm}}\right]}^{T}$. $R_{1f}$ and $R_{2f}$ are the longitudinal and transversal relaxation rates of the free pool, respectively, $k_{\mathrm{mf}}$ and $k_{\mathrm{fm}}$ are the exchange rates from macromolecular to free pool and free to macromolecular pool, respectively, and the pool‐size‐ratio $F=M_{0m}/M_{0f}$ describes the ratio of the free pool $M_{0f}$ and the macromolecular pool $M_{0m}$. Within the range n·TR<t<(n+1)·TR, Equation (3) results in a *first‐order linear inhomogeneous matrix ODE*

(4) $$\begin{array}{c} \frac{dM(t^{\prime })}{dt^{\prime }}=\xi_{1}M\left(t^{\prime }\right)+\xi_{2}M_{0} \end{array}$$

as the relaxation and exchange matrix $\xi_{1}(t^{\prime })=\xi_{1}$ is time independent. The solution to this *first‐order linear inhomogeneous matrix ODE* is given by

(5) $$\begin{array}{c} M\left(t\right)=e^{\xi_{1}t}M\left(t=0\right)+{\xi_{1}}^{‐1}\left(e^{\xi_{1}t}‐I\right)\xi_{2}M_{0} \end{array}$$

For repeated iterations of the pulse (n→∞), the magnetization reaches a dynamic steady state, satisfying the condition

(6) $$\begin{array}{c} M^{‐}\left(n+1\right)=R_{z}M^{‐}\left(n\right)\Leftrightarrow M^{+}\left(n+1\right)=R_{z}M^{+}\left(n\right) \end{array}$$

where the rotation matrix $R_{z}:=R_{z}(\Phi =180^{\circ })$ represents the change in sign of the flip angle after each iteration

(7) $$\begin{array}{c} R_{z}\left(\Phi =180^{\circ }\right)=\left[\begin{array}{cccc} ‐1 & \;0\; & \;\;0\; & \;\;0\;\;\\ \;0 & ‐1\; & \;\;0\; & \;\;0\;\;\\ \;0 & \;0\; & \;\;1\; & \;\;0\;\;\\ \;0 & \;0\; & \;\;0\; & \;\;1\;\; \end{array}\right] \end{array}$$

The magnetization during one pulse cycle (Epochs I and II), can be modeled by combining Equations (2) and (5). This allows one to relate the magnetization before the (n+1)th pulse to the magnetization before the nth pulse

(8) $$\begin{array}{c} M{\left(n+1\right)}^{‐}=e^{\xi_{1}TR‐I}R_{x}M{\left(n\right)}^{‐}+{\xi_{1}}^{‐1}\left(e^{\xi_{1}TR}‐I\right)\xi_{2}M_{0} \end{array}$$

This equation can be solved under the dynamic steady‐state condition (Equation 6) for n→∞ with the Ansatz

(9) $$\begin{array}{c} R_{z}M{\left(n\right)}^{‐}=M{\left(n+1\right)}^{‐} \end{array}$$

(10) $$\begin{array}{c} \Leftrightarrow \;R_{z}M{\left(n\right)}^{‐}=e^{\xi_{1}TR‐I}R_{x}M{\left(n\right)}^{‐}+{\xi_{1}}^{‐1}\left(e^{\xi_{1}TR}‐I\right)\xi_{2}M_{0} \end{array}$$

(11) $$\begin{array}{c} \Leftrightarrow \;M{\left(n\right)}^{‐}={\left(R_{z}‐e^{\xi_{1}TR}R_{x}\right)}^{‐1}{\xi_{1}}^{‐1}\left(e^{\xi_{1}TR}‐I\right)\xi_{2}M_{0} \end{array}$$

resulting in the solution at steady state

(12) $$\begin{array}{c} M_{\mathrm{SS}}=M{\left(n\to \infty \right)}^{+}=R_{x}{\left(R_{z}‐e^{\xi_{1}TR}R_{x}\right)}^{‐1}{\xi_{1}}^{‐1}\left(e^{\xi_{1}TR}‐I\right)\xi_{2}M_{0} \end{array}$$

The operator $R_{x}(\alpha ,t)$ represents an instant rotation of the magnetization in the free pool. This assumption is commonly used in MRI, but leads to an overestimation of transverse relaxation during excitation.^4^, ^5^ Throughout the rotation caused by a pulse of finite pulse duration, the magnetization spends a period when it has parallel alignment with the static magnetic field, that is, its equilibrium orientation. This reduces the transverse relaxation, which can be accounted for by the correction suggested by Bieri for the one‐pool model^4^

(13) $$\begin{array}{c} R_{2}\to \tilde{R_{2}}=\left(1‐\zeta \frac{T_{\mathrm{RFE}}}{\mathrm{TR}}\right)R_{2},\;\;\forall \;T_{\mathrm{RFE}}>0 \end{array}$$

with

(14) $$\begin{array}{c} \zeta \approx 0.68‐0.125\left(1+\frac{T_{\mathrm{RFE}}}{\mathrm{TR}}\right)\frac{R_{1}}{R_{2}} \end{array}$$

where the hard pulse time equivalent $T_{\mathrm{RFE}}$ is a pulse‐shape‐dependent constant. While this constant has previously been derived for Gaussian pulse shapes, here we present a solution for sinc pulse shapes (details in Appendix), as these are commonly used in qMT bSSFP

(15) $$\begin{array}{c} T_{\mathrm{RFE}}=\left\{\begin{array}{cc} T_{\mathrm{RF}} & \text{for hard pulse (by definition)}\\ 1.20\cdot \frac{T_{\mathrm{RF}}}{\mathrm{TBW}} & \text{for Gaussian pulse (proof in [4])}\\ \frac{4T_{\mathrm{RF}}}{TBW\pi }\frac{1‐\cos\left(\frac{\pi TBW}{2}\right)}{\text{Si}\left(\frac{\pi TBW}{2}\right)} & \text{for sinc pulse (proof in Appendix)} \end{array}\right. \end{array}$$

Here, Si denotes the sine integral defined in Equation (A7). The correction accounts for the overestimation of transverse relaxation during excitation and therefore considers the finite pulse duration; the derivation to the correction given by Equation (13) can be found at Ref. [4].

As the finite pulse duration correction only affects the transverse magnetization, which is generally neglected in the macromolecular pool, the two‐pool model can be corrected by transforming $R_{2}$ within the matrix $\tilde{\xi_{1}}=\xi_{1}(R_{2f}\to {\tilde{R}}_{2f})$

(16) $$\begin{array}{c} {\tilde{\xi }}_{1}=\left[\begin{array}{cccc} ‐\left(1‐\zeta \frac{T_{\mathrm{RFE}}}{\mathrm{TR}}\right)R_{2f} & 0 & 0 & 0\\ 0 & ‐\left(1‐\zeta \frac{T_{\mathrm{RFE}}}{\mathrm{TR}}\right)R_{2f} & \omega_{1} & 0\\ 0 & ‐\omega_{1} & ‐\left(R_{1f}+k_{\mathrm{fm}}\right) & k_{\mathrm{mf}}\\ 0 & 0 & k_{\mathrm{fm}} & ‐\left(R_{1m}+k_{\mathrm{fm}}\right) \end{array}\right] \end{array}$$

As $M_{\mathrm{xf}}$ is decoupled from the other components, the coupled equations can be reduced to $M={\left[M_{\mathrm{yf}},M_{\mathrm{zf}},M_{\mathrm{zm}}\right]}^{T}$. This leads to the corrected steady‐state solution for bSSFP in matrix notation, taking into account finite pulse duration effects and concurrent magnetization exchange and relaxation

(17) $$\begin{array}{c} M_{\mathrm{SS}}={\tilde{R}}_{x}{\left(R_{z}‐e^{{\tilde{\xi }}_{1}TR}{\tilde{R}}_{x}\right)}^{‐1}{\tilde{\xi }}_{1}^{‐1}\left(e^{{\tilde{\xi }}_{1}TR}‐I\right)\xi_{2}M_{0} \end{array}$$

Note that while Equation (17) describes the magnetization as a whole, experimentally only the transverse component of the free pool $M_{\mathrm{yf}}$ is measured.

## 3 METHODS

### 3.1 Numerical studies

To validate the analytical solution, simulations were performed by numerically solving the Bloch‐McConnell Equations for typical brain tissue parameters (Table 1).

**TABLE 1:** Typical qMT tissue parameters for different areas of the brain, taken from Refs. [3, 19]

Tissue	*F* (%)	$k_{\mathrm{mf}}$ ($s^{‐1}$)	$R_{1f}$ ($s^{‐1}$)	$T_{2f}$ (ms)
White matter	11	10	0.9	42
Gray matter	6	18	0.8	74
MS lesion	3	8	0.5	43

*Note*: $T_{2f}$: longitudinal relaxation time of the free pool.

Similar to the originally proposed acquisition, the flip angle α and the pulse duration $T_{\mathrm{RF}}$ have been varied while setting all other acquisition parameters constant. As suggested in the original paper by Gloor,^1^ an on‐resonance (Δω=0) sinc pulse shape has been chosen for excitation (Equation A2).

The effect of the sinc pulse on the free‐pool magnetization, given by $\omega_{1}$, has been simulated based on

(18) $$\begin{array}{c} \omega_{1}=\gamma B_{1}=\left\{\begin{array}{cc} \gamma A\left(\alpha ,t_{0}\right)\text{sinc}(\frac{\pi t}{t_{0}}) & \;\;\text{for}\;\;nTR‐T_{\mathrm{RF}}<t<nTR+T_{\mathrm{RF}}\\ 0 & \;\;\text{elsewhere} \end{array}\right. \end{array}$$

where the amplitude of each pulse has been calculated according to

(19) $$\begin{array}{c} A\left(\alpha ,t_{0}\right)=2\pi \alpha {\left(360^{\circ }\gamma \int_{‐T_{\mathrm{RF}}/2}^{T_{\mathrm{RF}}/2}\text{sinc}\left(\frac{\pi t}{t_{0}}\right)dt\right)}^{‐1} \end{array}$$

and the half‐width of the central lobe $t_{0}$ is related to $T_{\mathrm{RF}}$ and *TBW* according to Equation (A3). Due to its superior performance in tissue,^10^ a super‐Lorentzian lineshape has been chosen for absorption according to

(20) $$\begin{array}{c} R_{\mathrm{RF}}=\left\{\begin{array}{cc} \pi \gamma^{2}B_{1}^{2}g_{m}\left(\Delta \omega ,T_{2m}\right) & \;\;\text{for}\;\;nTR‐T_{\mathrm{RF}}<t<nTR+T_{\mathrm{RF}}\\ 0 & \;\;\text{elsewhere} \end{array}\right. \end{array}$$

for the nth iteration n∈{1,2,…,N} and

(21) $$\begin{array}{c} g_{m}\left(\Delta \omega ,T_{2m}\right)=\sqrt{\frac{\pi }{2}}\int_{0}^{\frac{\pi }{2}}\frac{T_{2m}}{|3u^{2}‐1|}\exp\left(‐2{\left(\frac{2\pi \Delta \omega T_{2m}}{3u^{2}‐1}\right)}^{2}\right)du \end{array}$$

Note that the super‐Lorentzian absorption lineshape has a singularity at $g_{m}\left(\Delta \omega =0\right)$. Analogous to previous studies,^1^, ^11^ the absorption lineshape has been extrapolated from 1 kHz to the asymptotic limit, resulting in $g_{m}\left(\Delta \omega \to 0\right)=1.4e‐5\;\mathrm{seconds}$ for which a constant $T_{2m}=12\;\mu s$ has been assumed.

### 3.2 In vivo studies

In addition to the simulations, the performance of the refined signal equation in comparison to the original one of Gloor et al^1^ was investigated in previously acquired human brain data. The images used in this work have been taken from an open source publication by Cabana et al.^12^ They were acquired for a single volunteer at 1.5 T (Siemens Healthineers, Erlangen, Germany) using the standard protocol of varied flip angles and pulse durations, suggested in the original publication on qMT bSSFP.^1^ The bSSFP acquisition parameters were varied as follows:

- Eight bSSFP sequences with constant flip angle $\alpha =35^{\circ }$ and varied pulse duration $T_{\mathrm{RF}}=0.23$, 0.3, 0.4, 0.58, 0.84, 1.2, 1.6 and 2.1 ms.
- Eight bSSFP sequences with constant pulse duration $T_{\mathrm{RF}}=0.27\;\mathrm{ms}$ and varied flip angle $\alpha =5^{\circ }$, $10^{\circ }$, $15^{\circ }$, $20^{\circ }$, $25^{\circ }$, $30^{\circ }$, $35^{\circ }$, $40^{\circ }$.

The repetition time *TR* of each single sequence was chosen such that $T_{d}=TR‐T_{\mathrm{RF}}=2.7\;\mathrm{ms}$ remained constant and a sinc‐shaped RF pulse of TBW=2.7 was used for excitation. A field‐of‐view (FOV) of 256×256×32mm with acquisition matrix 128×128×16 was selected. Additionally, $T_{1}$ maps were acquired using 2 SPGR sequences with TR/TE=9.8ms/4.77ms, bandwidth = 140 Hz/Pixel and varying flip angles of $\alpha =4^{\circ }$ and $\alpha =15^{\circ }$ according to the DESPOT1 method.^1^, ^13^

### 3.3 Quantitative MT parameter analyis

In order to determine the qMT parameters in each voxel, the qMT bSSFP signal equation was fitted to the acquired steady‐state magnetization by means of a nonlinear least‐squares fit. Both refined and original qMT bSSFP signal equations are dependent on five parameters: *F*, $k_{\mathrm{mf}}$, $T_{1f}$, $T_{2f}$ and $T_{1m}$. However, the additional acquisition of $T_{1f}$ allows that parameter to be fixed and $T_{1m}$ can be set equal to $T_{1f}$ due to its insensitivity to the magnetization.^1^, ^14^, ^15^ Thus, the remaining parameters *F*, $k_{\mathrm{mf}}$ and $T_{2f}$ were fitted on a voxel‐by‐voxel basis within the ranges 0.01≤F≤30%, $0.0001\leq k_{\mathrm{mf}}\leq 100s^{‐1}$ and $0.01\leq T_{2f}\leq 0.2\;\mathrm{seconds}$ using the starting points $\hat{F}=10\%$, ${\hat{k}}_{\mathrm{mf}}=30s^{‐1}$ and ${\hat{T}}_{2f}=0.04\;\mathrm{seconds}$. $M_{0f}$ has been set to one as has been done previously.^16^ All computations were performed in Matlab (MathWorks, Natick, MA) and code has been partially taken from the qMRlab toolbox.^12^ A nonparametric Wilcoxon signed‐rank test was used for statistical testing.

## 4 RESULTS

### 4.1 Numerical studies

**FIGURE 1:**
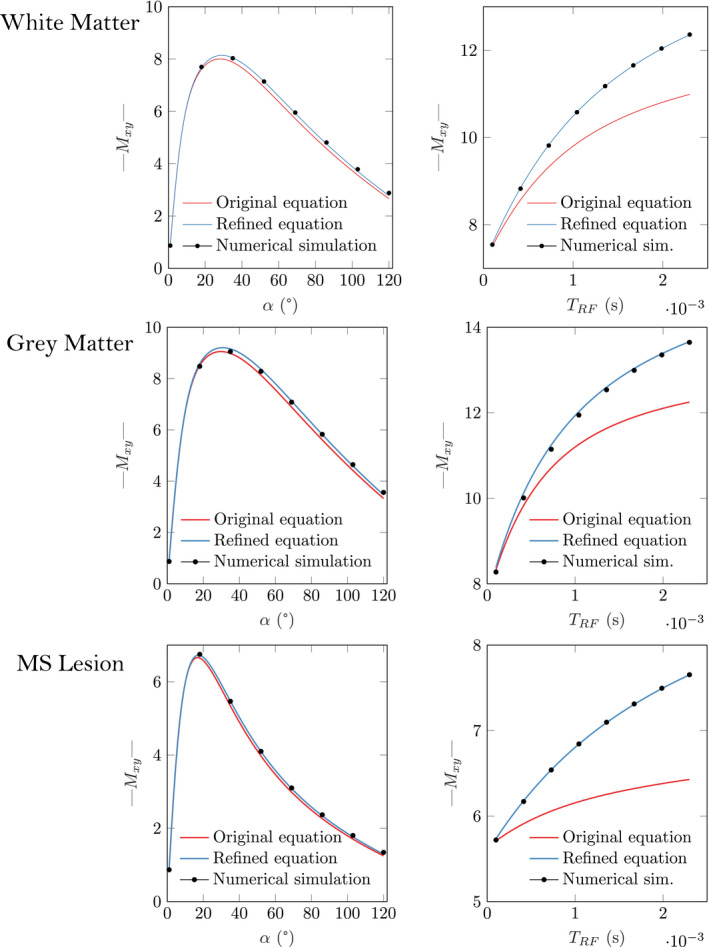
Original (red) and refined (blue) qMT bSSFP signal equation, next to the numerically simulated data (black dots), in a standard acquisition scheme of varied flip angles (left) and pulse durations (right). The plot used the parameters in Table 1 and constant values are $\alpha =35^{\circ }$ and $T_{\mathrm{RF}}=0.3\;\mathrm{ms}$

Figure 1 shows the original (red) and refined (blue) qMT bSSFP signal equations, along with the numerically simulated data (black dots) for a standard acquisition scheme for typical brain tissue parameters (Table 1). The simulation has been performed for a standard acquisition scheme of varied flip angles (left) and pulse durations (right). Taking the numerical simulation as the mathematical ground truth, Figure 1 shows a bias in the original signal equation of up to 11.1% at $T_{\mathrm{RF}}=2.3\;\mathrm{ms}$. The maximum bias of both original and refined signal equations for the different tissue parameters are summarized in Table 2. While the original model is affected by a maximal bias of up to 20.3% (for an MS lesion), the refined signal equation describes the numerically simulated data with a bias <1% in all three tissues.

**TABLE 2:** Maximal percentage deviation of analytical signal equations from the numerical simulation $\Delta M_{y,\text{max.}}=\max\left[\left(M_{y,\text{simulation}}‐\Delta M_{y,\text{analytical}}\right)/M_{y,\text{simulation}}\right]$ in the standard protocol of varied flip angles and pulse durations

Tissue	Original bias $\Delta M_{y,\text{max.}}$	Refined bias $\Delta M_{y,\text{max.}}$
White matter	11.1%	0.7%
Gray matter	7.4%	0.3%
MS lesion	20.3%	0.4%

*Notes*: The maximum has been calculated for $T_{\mathrm{RF}}$ ranging from 0.2 to 2.3 ms, α ranging from $5^{\circ }$ to $40^{\circ }$, fixed $t_{d}=TR‐T_{\mathrm{RF}}=2\;\mathrm{ms}$, TBW=2 and the qMT parameters of Table 1.

### 4.2 In vivo studies

**FIGURE 2:**
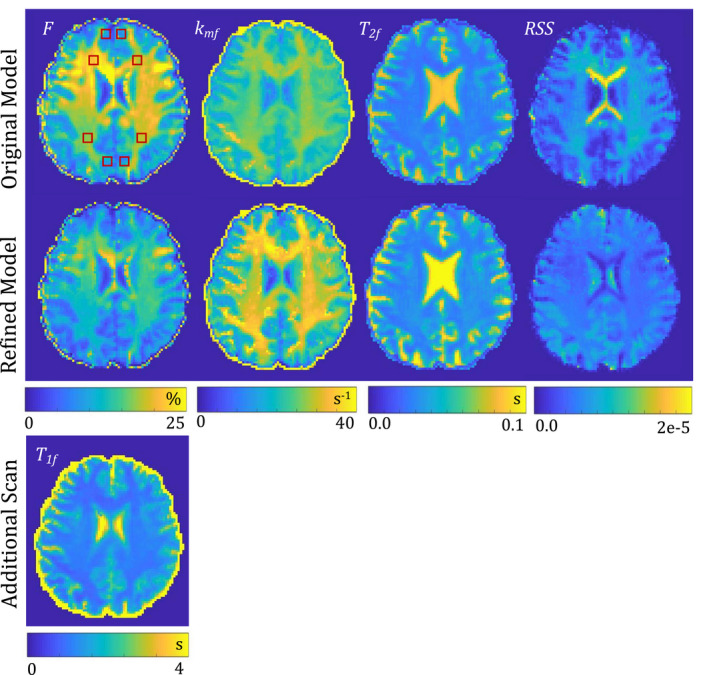
QMT parameter maps of a healthy brain, as analyzed by the original (top row) and the refined (bottom row) model. In addition, the residual sum of squares (RSS) of the fit and $T_{1f}$ maps are displayed. The qMT parameters, fitted for each voxel, are as follows: pool‐size‐ratio *F*, exchange rate $k_{\mathrm{mf}}$ and relaxation time of the free pool $T_{2f}$. Red squares mark ROIs

The resulting qMT parameter maps of a voxelwise least‐squares fit on the human data are shown in Figure 2. The analysis was performed using the original and the refined signal equations. The mean values of all fitted qMT parameters within regions of interest (ROIs) in gray and white matter are listed in Table 3.

**TABLE 3:** Fitted qMT parameters within healthy brain structures, as determined by the original and the refined model

Tissue	$F_{\text{org.}}$ (%)	$F_{\text{ref.}}$ (%)	$k_{mf,\text{org.}}$ ($s^{‐1}$)	$k_{mf,\text{ref.}}$ ($s^{‐1}$)	$T_{2f,\text{org.}}$ (ms)	$T_{2f,\text{ref.}}$ (ms)
Frontal WM	18.4 ± 1.2	12.7 ± 0.8	28.2 ± 0.6	35.6 ± 1.3	31 ± 5	27 ± 4
Frontal GM	8.6 ± 1.4	6.5 ± 1.1	22.2 ± 2.8	22.9 ± 4.9	62 ± 11	54 ± 9
Occipital WM	19.4 ± 0.8	14.1 ± 0.5	27.2 ± 0.8	33.1 ± 1.3	32 ± 2	28 ± 2
Occipital GM	7.9 ± 0.6	5.9 ± 0.7	24.0 ± 3.7	25.2 ± 8.6	32 ± 6	29 ± 5

*Notes*: Shown are the mean ± standard deviation (SD) values across the ROIs. WM: white matter, GM: gray matter.

For the clinically relevant pool‐size‐ratio *F*, a significant difference (*P* < .001) between both models can be observed within ROIs in white and gray matter. Compared to the original model ($F_{\text{org.}}$), *F* decreased in the refined model ($F_{\text{ref.}}$) from 18.4 ± 1.2%/19.4 ± 0.8% to 12.7 ± 0.8%/14.1 ± 0.5% in frontal/occipital white matter, respectively. Similarly, *F* decreased from 8.6 ± 1.4% to 6.5 ± 1.1% and 7.9 ± 0.6% to 5.9 ± 0.7% in frontal and occipital gray matter, respectively. The difference between the estimates of the refined and original signal equation is statistically larger ( P<.001) in white matter compared to gray matter.

The exchange rate, analyzed by the refined model, $k_{mf,\text{ref.}}$ differs from the original model $k_{mf,\text{org.}}$ only in white matter (P<.001); no statistically significant difference has been found in gray matter (*P* = .25 and *P* = .49 in frontal and occipital gray matter, respectively). The refined model results in statistically lower transversal relaxation rates in the free pool $T_{2f}$ (P<.001), with differences ranging from 9%‐13% in all four ROIs.

The mean of the residual sum of squares (RSS) over all voxels is 7% lower in the refined model compared to the original one.

### 4.3 Finite pulse duration correction for sinc shape

**FIGURE 3:**
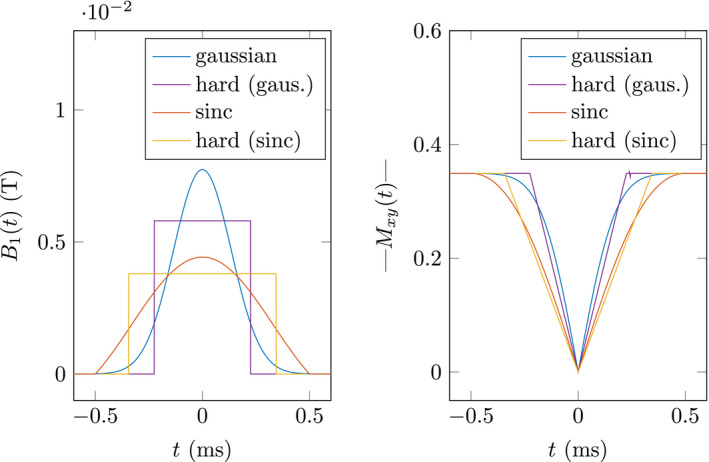
Illustration of hard pulse equivalent for Gaussian and sinc pulse shapes. The RF pulse (left) and the corresponding transverse magnetization trajectory (right) are plotted for both pulse shapes and their hard pulse equivalent. Both pulse shapes are plotted for $\alpha =40^{\circ }$, $T_{\mathrm{RF}}=1\;\mathrm{ms}$. To allow for a clear distinction, TBW=2 for sinc pulse and TBW=2.6 for the Gaussian pulse

Figure 3 (left) shows a Gaussian and a sinc pulse of similar flip angle together with their respective hard pulse equivalents. The contributing magnetizations in Figure 3 (right) show, that the areas enclosed under the curves are equal for each pulse and its respective hard pulse equivalent. This illustrates the definition of the hard pulse equivalent (Equation A4).

Exemplary values of $T_{\mathrm{RFE}}$ for the different pulse shapes are listed in Table 4, showing significant differences for the same *TBW*. The hard pulse equivalent duration approaches zero for TBW∈{4,8,12,…} in the sinc pulse. This implies that the correction becomes unnecessary in this case (ie, ${\tilde{R}}_{2}=R_{2}$), as the correction term for finite pulse durations directly correlates with the hard pulse equivalent duration $\Delta R_{2f}\propto T_{\mathrm{RFE}}$ (Equation 13).

**TABLE 4:** Exemplary hard pulse equivalent duration $T_{\mathrm{RFE}}$ of Gaussian, sinc and hard pulse shapes for different *TBW*, resulting from Equation (15)

*TBW*	Sinc pulse $T_{\mathrm{RFE}}$	Gaussian pulse $T_{\mathrm{RFE}}$	Hard pulse $T_{\mathrm{RFE}}$
2	0.69 $T_{\mathrm{RF}}$	0.60 $T_{\mathrm{RF}}$	1.00 $T_{\mathrm{RF}}$
3	0.26 $T_{\mathrm{RF}}$	0.40 $T_{\mathrm{RF}}$	1.00 $T_{\mathrm{RF}}$
4	0	0.30 $T_{\mathrm{RF}}$	1.00 $T_{\mathrm{RF}}$

The derived relation (Equation 15) allows correction for the $T_{2}$‐bias in the bSSFP signal equation (both standard and qMT specific) when using sinc pulse shapes. The bias, induced by the overestimation of transversal relaxation during excitation, can be corrected by substituting $R_{2}\to {\tilde{R}}_{2}$ in the original signal equation. In the case of a sinc pulse shape, the correction factor is as follows:

(22) $$\begin{array}{c} {\tilde{R}}_{2}=R_{2f}‐R_{2f}\frac{4\zeta T_{\mathrm{RF}}}{TBW\pi TR}\frac{1‐\cos(\frac{\pi TBW}{2})}{\text{Si}(\frac{\pi TBW}{2})} \end{array}$$

where

(23) $$\begin{array}{c} \zeta \approx 0.68‐0.125(1+\frac{4T_{\mathrm{RF}}}{TBW\pi TR}\frac{1‐\cos(\frac{\pi TBW}{2})}{\text{Si}(\frac{\pi TBW}{2})})\frac{R_{1}}{R_{2}} \end{array}$$

## 5 DISCUSSION

The simulations have shown that the original qMT bSSFP signal equation is biased by the assumptions made in its derivation (firstly separation of exchange and relaxation and secondly instantaneous rotation of the RF pulse). This bias has been seen to be tissue dependant, amounting to deviations of up to 7% and 11% in white and gray matter of healthy brain tissue and exceeding 20% in an MS lesion. The tissue dependence is expected, as the bias linearly depends on the relaxation time ratio $\frac{T_{2}}{T_{1}}$ (Equation 13), which varies amongst different tissue types. Furthermore, the bias has been shown to increase at higher pulse durations $T_{\mathrm{RF}}$. This reflects the fact that while the assumption of an instantaneous rotation by the RF pulse might be sufficient for short pulse durations, it is increasingly violated at longer $T_{\mathrm{RF}}$. In qMT bSSFP, this bias is particularly strong, as the acquisition involves long $T_{\mathrm{RF}}$ relative to *TR*.^1^ The bias is passed on to the qMT parameters, as they are determined by fitting the signal equation to the acquired data.

To address this, the suggested refined signal equation for qMT bSSFP (Equation 17) has been derived, accounting for the assumptions made in the original model, and describes the simulated data with minimal bias (<1%).

The comparison of original and refined signal equations in‐vivo shows significant differences between the resulting qMT parameters (24%‐31% for pool‐size‐ratio, 0%‐21% for exchange rate and 9%‐13% for transversal relaxation time). In agreement with the simulation results, the difference between qMT parameters, determined by the original and refined model, is significantly greater in white matter compared to gray matter in in‐vivo brain tissue data. This is in agreement with the theoretically predicted $\frac{T_{2}}{T_{1}}$ dependency of the bias (Equation 13).

In previous studies of different qMT modalities, pool‐size‐ratios in the range of 10%‐16% and 3%‐8% have been reported in white and gray matter structures, respectively.^17^, ^18^, ^19^, ^20^, ^21^ The pool‐size‐ratios, determined by the original model in this work, exceed the previously reported range in white matter (18.4 ± 1.2%, 19.3 ± 0.8%) and approach the upper limit in gray matter (8.6 ± 1.2%, 7.9 ± 0.6%). In contrast, the refined model estimates of the pool‐size‐ratio are in good agreement with the findings in other studies in both white matter structures (12.7 ± 0.8%, 14.1 ± 0.5%) and gray matter structures (6.5 ± 1.1%, 5.9 ± 0.7%). This indicates that the refined model outperforms the original one not only in simulation, but also in‐vivo. This conclusion is further supported by the significantly lower RSS found in the fits of the refined model compared to the original one. The wide range of previously reported exchange rates in different qMT methodologies 10‐$40\;s^{‐1}$ includes the results in both original and refined signal equations in this work.

The pool‐size‐ratios determined by qMT bSSFP in Ref. [1] are 13%‐16% and 6%‐7% for white and gray matter structures, respectively. Although they fall at the upper end of previous findings, they are lower than the values found with the original model in this work. The reason for reduced biases in the original findings^1^ can be explained by means of the pulse shape analysis, established in Section 3.3. While the parameters of the original findings have been acquired with a TBW=2.7, in this work a TBW=2 has been used. Their respective hard pulse equivalent durations, which correlate with the bias $\Delta R_{2}\propto T_{\mathrm{RFE}}$, differ by 42% $\left(\frac{T_{\mathrm{RFE}}\left(TBW=2.0\right)}{T_{\mathrm{RFE}}\left(TBW=2.7\right)}=0.58\right)$. This implies a reduction of the bias in the original acquisition scheme for a TBW=2.7 and explains why bias is reduced in the original publication.^1^ While the bias is only reduced and not removed, much higher biases are expected for a TBW≤2.5. Alternatively, the refined signal equation allows for a general solution with accurate parameter estimation for a wide range of *TBW*.

Additionally, the derived Equation (15) predicts that the bias oscillates for varying *TBW* and even approaches zero for a TBW∈{4,8,12,…}. The physical explanation for the oscillation lies in the sinc‐shape‐specific side lobes. These side lobes cause a temporary increased deflection of magnetization from the equilibrium alignment, for which transverse relaxation is underestimated. The underestimation, induced by the negative side lobes, counterbalances the overestimation, resulting from the main lobe. Therefore, the bias in quantitative bSSFP methods (qMT and non‐qMT) can be removed by choosing an appropriate time‐bandwidth product without using the correction given by Equation (13). This might be useful for applications where the correction is inaccurate due to strong magnetic field inhomogeneities, such as is the case at high magnetic field strengths.

Recent work by Wood et al^22^ has demonstrated that the PLANET method^23^, ^24^, ^25^ for phase‐cycled bSSFP can be applied to qMT at higher field strengths to derive qMT parameter estimates free from banding artefacts. However, Wood et al^22^ utilized the signal model from Gloor et al,^1^ which translated into increased errors in their parameter estimation, particularly in white matter. We hypothesize that combining the method from Wood et al^22^ with our methods here would provide increased accuracy, leading to a method which can produce qMT parameter estimates quickly over all clinical field strengths. However, this is beyond the scope of this paper, and is left for future work.

## 6 CONCLUSION

A new signal equation for qMT bSSFP was derived, which incorporates both finite pulse effects and simultaneous magnetization exchange and relaxation. Numerical simulations of the Bloch‐McConnell equations showed that the original signal equation is significantly biased in typical brain tissue structures (by 7%‐20%). By contrast, the new signal equation outperforms the original one with minimal bias (< 1%). The practicality of the new signal equation was demonstrated using in vivo data and it is shown that the bias of the original signal equation strongly affects the acquired qMT parameters in human brain structures, with differences in the clinically relevant pool‐size‐ratio of up to 31%. Particularly high biases of the original signal equation are expected in an MS lesion within diseased brain tissue (due to a low $\frac{T_{2f}}{T_{1f}}$‐ratio), demanding a more accurate model for clinical applications. Therefore, the refined signal equation is recommended for accurate qMT parameter estimation in healthy and diseased brain tissue, especially when using a TBW≤2.5.

## CONFLICT OF INTEREST

P.J. is the Editor‐in‐Chief of Magnetic Resonance in Medicine. In line with COPE guidelines, he recused himself from all involvement in the review process of this paper, which was handled by an Associate Editor. He and the other authors have no access to the identity of the reviewers.

## APPENDIX A FINITE PULSE DURATION CORRECTION FOR SINC SHAPE

### A.1

As shown by Bieri,^4^, ^5^ the overestimation of transverse relaxation in bSSFP can be corrected by means of the substitution of $R_{2}\to {\tilde{R}}_{2}$ according to Equation (13). This correction has been derived under the assumption of excitation by a constant RF magnetic field (ie, a hard pulse). By means of the hard pulse equivalent duration $T_{\mathrm{RFE}}$, the correction can be transferred to different pulse shapes

(A1)
$$\begin{array}{c} T_{\mathrm{RFE}}\left\langle B\right\rangle =\int B_{1}\left(t\right)dt \end{array}$$

where ⟨B⟩ is the mean $B_{1}$ amplitude. This relation has previously been solved for a Gaussian pulse shape, resulting in $T_{\mathrm{RFE}}=1.2\;\frac{T_{\mathrm{RF}}}{\mathrm{TR}}$.^4^ In this section, Ansatz A1 is solved for a sinc pulse shape.

Consider a sinc pulse of form

(A2)
$$\begin{array}{c} B_{1}\left(t\right)=\left\{\begin{array}{cc} A\;\text{sinc}\left(\frac{\pi t}{t_{0}}\right) & \;\;\text{for}‐N_{L}t_{0}\leq t\leq N_{R}t_{0}\\ 0 & \;\;\text{elsewhere} \end{array}\right. \end{array}$$

where $t_{0}$ is the half‐width of the central lobe, *A* is the amplitude of the pulse and *t* is the time. $N_{L}$ and $N_{R}$ are the numbers of zero‐crossings of the sinc pulse to the left and right of the central peak, respectively (if symmetric: $N_{L}=N_{R}$). The full width at half maximum (FWHM) of a sinc pulse can be approximated by $\text{FWHM}=\Delta f\approx \frac{1}{t_{0}}$ ^26^ and the time bandwidth product is defined as

(A3)
$$\begin{array}{c} TBW=N_{L}+N_{R}=N=T_{\mathrm{RF}}\Delta f\approx \frac{T_{\mathrm{RF}}}{t_{0}} \end{array}$$

with the number of total zero‐crossings *N*.

As shown by Bieri,^4^ Ansatz A1 leads to a relation between $T_{\mathrm{RFE}}$ and magnetization

(A4)
$$\begin{array}{c} {\left\langle M_{\mathrm{xy}}\right\rangle }^{+}=(1‐\frac{T_{\mathrm{RFE}}}{2T_{\mathrm{RF}}})M_{\mathrm{xy}}^{‐} \end{array}$$

where $M_{\mathrm{xy}}^{‐}$ is the magnetization before excitation and ${\left\langle M_{\mathrm{xy}}\right\rangle }^{+}$ is the time average magnetization during excitation. By calculating the pulse shape‐dependent ${\left\langle M_{\mathrm{xy}}\right\rangle }^{+}$, Equation (A4) can be used to find the hard pulse equivalent duration $T_{\mathrm{RFE}}$. For sufficiently small flip angles, the magnetization can be approximated to

(A5)
$$\begin{array}{c} M_{\mathrm{xy}}\left(t\right)\approx \frac{\left|\alpha (t)‐\alpha /2\right|}{\alpha /2}\cdot M_{\mathrm{xy}}^{‐}=|\gamma \int_{‐T_{\mathrm{RF}}/2}^{t}\frac{B_{1}\left(t^{\prime }\right)}{\alpha /2}dt^{\prime }‐1|\cdot M_{\mathrm{xy}}^{‐} \end{array}$$

where the the relations of flip angle $\alpha \left(t\right)=\int_{‐T_{\mathrm{RF}}/2}^{t}\omega_{1}\left(t^{\prime }\right)dt^{\prime }$ and $\omega_{1}\left(t\right)=\gamma B_{1}\left(t\right)$, have been used.

Using the symmetry property of the sinc function sinc(x)=sinc(‐x), the flip angle can be expressed as

(A6)
$$\begin{array}{c} \alpha =\gamma \int_{‐T_{\mathrm{RF}}/2}^{T_{\mathrm{RF}}/2}B_{1}\left(t\right)dt=2\gamma \int_{0}^{T_{\mathrm{RF}}/2}B_{1}\left(t\right)dt=2\gamma \frac{At_{0}}{\pi }\int_{0}^{\frac{T_{\mathrm{RF}}\pi }{2t_{0}}}\text{sinc}\left(\theta \right)d\theta \end{array}$$

where the substitution $\frac{\pi t}{t_{0}}=\theta$ has been used. The integral can be solved using the sine integral definition

(A7)
$$\begin{array}{c} \text{Si}\left(x\right)=\int_{0}^{x}\frac{\sin(\theta )}{\theta }d\theta \end{array}$$

leading to a relation between flip angle, pulse duration, and half‐bandwidth

(A8)
$$\begin{array}{c} \alpha =2\gamma \frac{At_{0}}{\pi }\;\text{Si}\left(\frac{T_{\mathrm{RF}}\pi }{2t_{0}}\right) \end{array}$$

Calculating the time average magnetization during excitation, and exploiting the symmetry around t=0, results in

(A9)
$$\begin{array}{c} {\left\langle M_{\mathrm{xy}}\right\rangle }^{+}=\frac{1}{T_{\mathrm{RF}}}\int_{‐T_{\mathrm{RF}}/2}^{T_{\mathrm{RF}}/2}M_{\mathrm{xy}}\left(t\right)dt=\frac{2}{T_{\mathrm{RF}}}\int_{0}^{T_{\mathrm{RF}}/2}\left|\gamma \int_{‐T_{\mathrm{RF}}/2}^{t}\frac{B_{1}\left(t^{\prime }\right)}{\alpha /2}dt^{\prime }‐1\right|\cdot M_{\mathrm{xy}}^{‐}dt \end{array}$$

The integral of $B_{1}\left(t\right)$ can be split $\forall \;t\in [0,T_{\mathrm{RF}}/2]$ into

(A10)
$$\begin{array}{c} \int_{‐T_{\mathrm{RF}}/2}^{t}B_{1}\left(t^{\prime }\right)dt^{\prime }=\int_{‐T_{\mathrm{RF}}/2}^{0}B_{1}\left(t^{\prime }\right)dt^{\prime }+\int_{0}^{t}B_{1}\left(t^{\prime }\right)dt^{\prime }=\frac{\alpha }{2\gamma }+\int_{0}^{t}B_{1}\left(t^{\prime }\right)dt^{\prime } \end{array}$$

where the definition of flip angle α and the symmetric nature of the sinc function have been used. This simplifies Equation (A9) to

(A11)
$$\begin{array}{c} \begin{array}{cc} {\left\langle M_{\mathrm{xy}}\right\rangle }^{+} & =\frac{2}{T_{\mathrm{RF}}}\int_{0}^{T_{\mathrm{RF}}/2}\left|\frac{2\gamma }{\alpha }\left(\frac{\alpha }{2\gamma }+\int_{0}^{t}B_{1}\left(t^{\prime }\right)dt^{\prime }\right)‐1\right|\cdot M_{\mathrm{xy}}^{‐}dt\\ & =\frac{4\gamma A}{T_{\mathrm{RF}}\alpha }\int_{0}^{T_{\mathrm{RF}}/2}\left|\int_{0}^{t}\text{sinc}\left(\frac{\pi t^{\prime }}{t_{0}}\right)dt^{\prime }\right|\cdot M_{\mathrm{xy}}^{‐}dt \end{array} \end{array}$$

Further substitutions and the use of the integral in Equation (A7) leads to

(A12)
$$\begin{array}{c} {\left\langle M_{\mathrm{xy}}\right\rangle }^{+}=\frac{4\gamma A}{T_{\mathrm{RF}}\alpha }\frac{t_{0}}{\pi }\int_{0}^{T_{\mathrm{RF}}/2}\left|\text{Si}\left(\frac{\pi t}{t_{0}}\right)\right|\cdot M_{\mathrm{xy}}^{‐}dt=\frac{4\gamma A}{T_{\mathrm{RF}}\alpha }{\left(\frac{t_{0}}{\pi }\right)}^{2}\int_{0}^{\frac{T_{\mathrm{RF}}\pi }{2t_{0}}}\left|\text{Si}(u)\right|\cdot M_{\mathrm{xy}}^{‐}du \end{array}$$

The integral of the sine integral Si(t) is found by means of partial integration

(A13)
$$\begin{array}{c} \int_{0}^{T}\text{Si}\left(t\right)dt=\int_{0}^{T}1\cdot \text{Si}\left(t\right)dt\overset{\text{PI}}{=}{\left[t\;\text{Si}(t)\right]}_{0}^{T}‐\int_{0}^{T}\sin\left(t\right)dt=T\;\text{Si}\left(T\right)+\cos\left(T\right)‐1 \end{array}$$

to yield

(A14)
$$\begin{array}{c} {\left\langle M_{\mathrm{xy}}\right\rangle }^{+}=\left[1+\frac{2}{TBW\pi }\frac{1}{\text{Si}\left(\frac{\pi TBW}{2}\right)}\left(\cos\left(\frac{\pi TBW}{2}\right)‐1\right)\right]M_{\mathrm{xy}}^{‐} \end{array}$$

where relation (A3) has been used.

Inserting the derived expression for the time average magnetization (A14) into Ansatz (A4) finally leads to the relation

(A15)
$$\begin{array}{c} T_{\mathrm{RFE}}=\frac{4T_{\mathrm{RF}}}{TBW\pi }\frac{1‐\cos(\frac{\pi TBW}{2})}{\text{Si}(\frac{\pi TBW}{2})} \end{array}$$

This relation allows for correction of overestimation of transversal relaxation in bSSFP, when using a sinc pulse for excitation. Analogous to the correction for Gaussian and hard pulse shapes, a substitution $R_{2}\to {\tilde{R}}_{2}\left(T_{\mathrm{RFE}}\right)$ corrects for the bias according to Equation (13).
